# Case Report: Loeffler endocarditis as a cause of left ventricular thrombosis in young women: a case study and literature review

**DOI:** 10.3389/fcvm.2024.1456788

**Published:** 2024-12-13

**Authors:** Yue Cui, Yugen Shi, Xiaojun Wang

**Affiliations:** Department of Cardiology, The First Affiliated Hospital of Shandong First Medical University, Shandong First Medical University, Shandong, China

**Keywords:** loeffler endocarditis, left ventricular thrombosis, hypereosinophilic syndrome, young patients, cerebral infarction

## Abstract

It is unusual for young patients without any underlying diseases to experience sudden cerebral infarction and heart failure. Here, we report a rare case of a 28-year-old female patient who presented with chest tightness and dizziness. Left ventricular thrombus formation and cardiac insufficiency were evident on echocardiogram, while multiple acute or subacute cerebral infarctions were visible on brain magnetic resonance imaging. We preliminarily determined that this was a different manifestation of the same disease. After investigating the cause, we diagnosed the patient with Loeffler endocarditis caused by idiopathic eosinophilia syndrome involving the heart. Although no endocardial biopsy was performed, this diagnosis was confirmed through cardiac magnetic resonance imaging (CMR). After drug treatment consisting of corticosteroids and anticoagulants, the eosinophil count decreased significantly; however, the thrombus did not completely disappear, as assessed in multiple follow-up echocardiogram sessions. Further exploration of the tissue composition of the patient's left ventricular mass suggested that the mass was a mixture of thrombus and eosinophilic granulation tissue. The addition of imatinib to the treatment plan had a good therapeutic effect, and the patient's left ventricular mass completely disappeared. Loeffler endocarditis progresses rapidly and requires early identification and intervention by clinicians. This case emphasizes that, despite the lack of an endocardial biopsy, the early diagnosis of Loeffler endocarditis can be made through CMR, while avoiding the occurrence of irreversible endocardial fibrosis. We also explored the nature of the patient's cardiac mass and proposed different insights. The nature of cardiac mass varies in different stages of Loeffler endocarditis, and individualized treatment strategies are needed.

## 1 Introduction

Hypereosinophilic syndrome (HES) includes a rare group of diseases defined as a persistently high eosinophil count (>1.5  ×  10^9^/L) and eosinophil-related organ damage (1). When a large number of eosinophils infiltrate the heart, the bioactive molecules produced by their degranulation can cause explosive myocarditis or chronic restrictive cardiomyopathy (Loeffler endocarditis). Loeffler endocarditis is characterized by the thickening of the endocardium of one or both ventricles, thrombus and fibrosis of the endocardium, and clinical manifestations of cardiac dysfunction such as chest tightness, wheezing, and fatigue (2). An endocardial biopsy is the gold standard for its diagnosis; however, owing to its invasiveness and difficulty with patient acceptance, cardiac magnetic resonance imaging (CMR) is a useful auxiliary approach (3). However, clear treatment guidelines for Loeffler endocarditis are lacking. Therefore, the current case report provides a framework for the diagnosis and treatment of Loeffler endocarditis and proposes different perspectives about ventricular masses.

## 2 Case description

**Figure 1:**
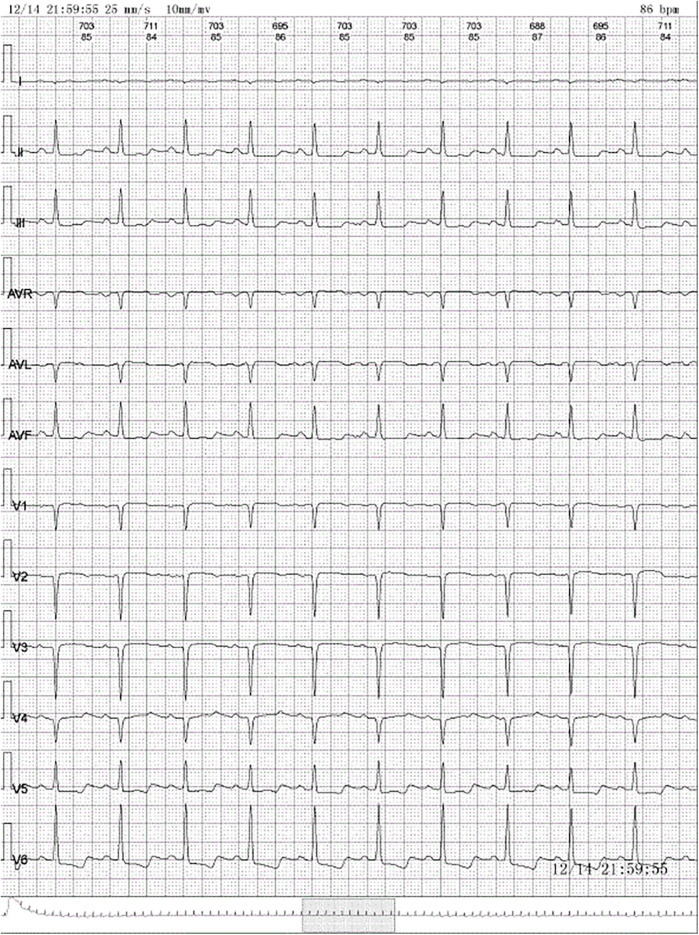
Electrocardiogram: normal sinus rhythm, ST-segment downward shift in leads II, III, and AV_F_ and QS in leads V1–V4.

A 28-year-old woman was admitted to the neurology department with dizziness and chest tightness that had persisted for half a month. A cranial computed tomography (CT) scan revealed no significant abnormalities. The electrocardiogram (ECG) showed ST-segment downward shift in leads II, III, and AV_F_, and QS in leads V1–V4 (Figure 1). She was advised to get sufficient rest and was scheduled for a follow-up visit. Over the next 20 days her chest tightness worsened. She needed to rest after walking only 200–300 m on flat ground and could not lie down comfortably. She sought medical attention from the cardiology department and underwent further investigations. Blood tests showed an elevated white blood cell count (14.92 × 10^9^/L; reference range: 3.5–9.5 × 10^9^/L) with increased neutrophil (6.78 × 10^9^/L; reference range: 1.8–6.3 × 10^9^/L) and eosinophil counts (4.5 × 10^9^/L; reference range: 0.02–0.52 × 10^9^/L). B-type natriuretic peptide level was elevated (840 pg/ml; reference range: 0–100 pg/ml), and lactate dehydrogenase level was high (591 U/L; reference range: 135–214 U/L). ECG again showed sinus tachycardia and ST-T changes.

Brain magnetic resonance imaging (MRI) revealed multiple acute or subacute cerebral infarctions in both cerebellar hemispheres. Cardiac ultrasound identified an enlarged left heart with an abnormal left ventricular structure (unclear whether congenital, hypertrophic cardiomyopathy, or incomplete myocardial densification). There was also mild mitral regurgitation, moderate tricuspid regurgitation, and pulmonary hypertension. She was diagnosed with acute cerebral infarction, respiratory infection, and heart failure. She received treatment with antibiotics, diuretics, and nutritional cardiac drugs (sodium phosphocreatine), following which her chest tightness improved. However, to further investigate the cause of her condition, she sought medical attention at our hospital.

Two main conditions are present: cerebral infarction and heart failure. It is important to determine if these are interconnected manifestations of a single underlying disease or entirely separate conditions. Cerebral infarction in young adults often has different causes from those in elder individuals. Atherosclerotic disease, a frequent risk factor in elderly populations, is less common in those under 30 years of age. Instead, the leading causes in young adults fall into two categories: cardiac causes (accounting for 20%–35% of cases) and non-cardiac right-to-left shunts (uncommon) (4). Cardiac causes typically involve emboli originating from sources like endocarditis, left ventricular thrombus, intracardiac right-to-left shunts, and atrial myxoma. Non-cardiac right-to-left shunts, such as pulmonary arteriovenous malformations and fistulas, are rare (4). The case patient was young and had no history of traditional cardiovascular risk factors. She also lacked a family history of stroke. Based on these factors, she did not belong to a typical high-risk group for stroke. Therefore, the possibility of atherosclerotic disease is low. Preliminary screening for pulmonary arteriovenous malformations and fistulas can be performed using cardiac ultrasound. For suspected patients, further enhanced CT can be performed to accurately evaluate vascular structure. The patient's preliminary screening for pulmonary arteriovenous malformations and fistulas using cardiac ultrasound did not consider this cause. After excluding the above factors, cardioembolic stroke was deemed the likely cause, but further investigation was needed to clarify the same.

**Figure 2:**
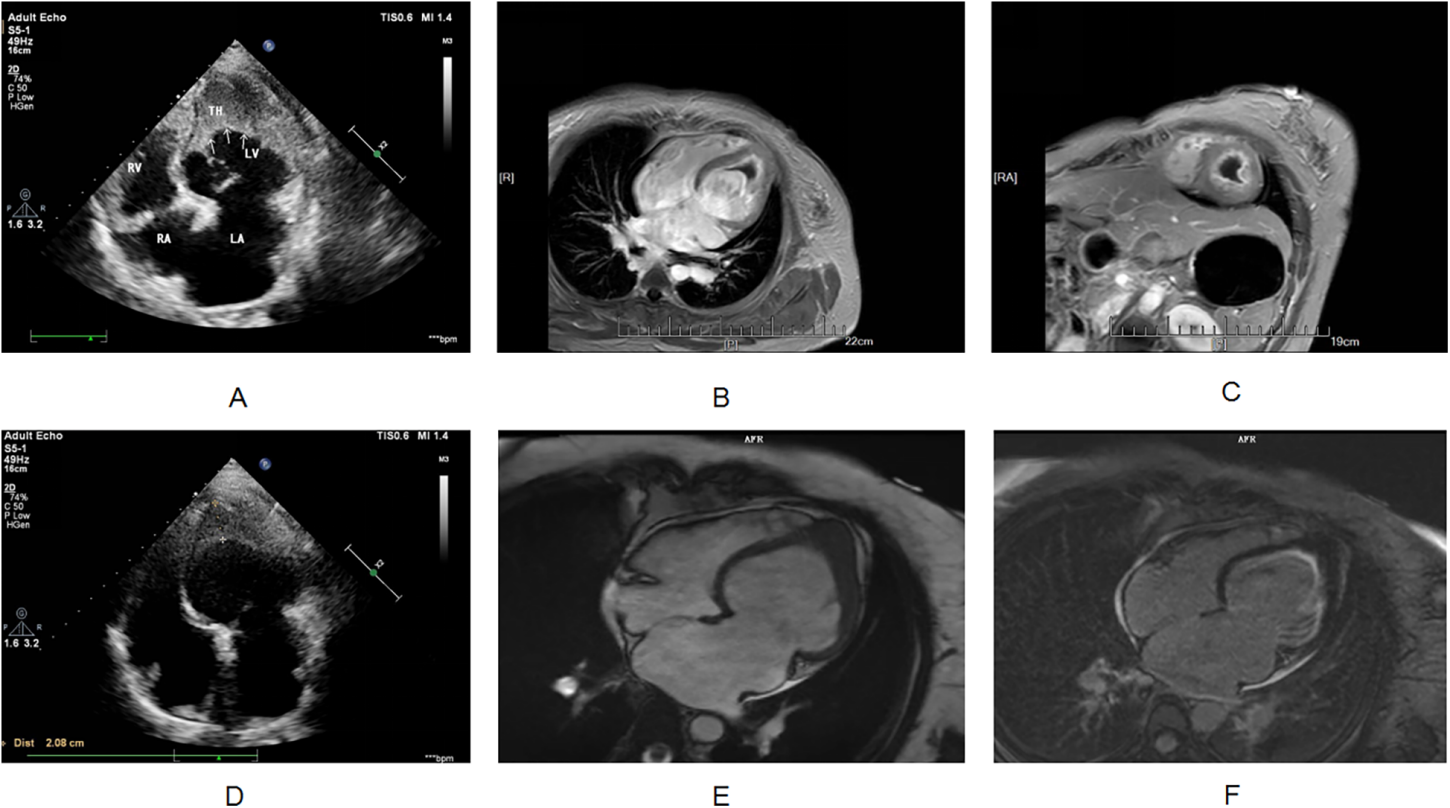
**(A)** Transthoracic echocardiography shows apical occlusion and numerous mid to high echogenic masses in the lower left ventricular cavity. **(B,C)** Cardiac magnetic resonance imaging shows occlusion of the apical segment of the left ventricle, and myocardial delayed enhancement imaging shows diffuse enhancement lesions beneath the endocardium of the left ventricle. The signal of the cardiac mass is not enhanced. **(D)** After hormone combined with warfarin treatment, apical occlusion was still observed on transthoracic echocardiography, and the number of hyperechoic masses in the lower left ventricular cavity decreased compared to before. **(E,F)** After one year of treatment with imatinib, cardiac magnetic resonance imaging showed apical occlusion, myocardial delayed enhancement imaging showed subendocardial enhancement in the left ventricle, and the cardiac mass completely disappeared.

The etiology of her heart failure remains under investigation. Potential causes included coronary artery disease, valvular heart disease, hypertension, primary or secondary cardiomyopathy, congenital heart disease, pericardial disease, and others (5). The above research indicated that the patient was less likely to have atherosclerotic disease and has no history of hypertension. Cardiac ultrasound showed abnormal left ventricular structure. It is unclear whether there is valvular heart disease, cardiomyopathy, pericardial disease or congenital heart disease. Therefore, the patient needed to undergo another echocardiogram and CMR, as necessary. The next step focused on investigating the causes of both the suspected cardioembolic event and heart failure. Echocardiogram revealed the following findings: left atrial anterior-posterior diameter 41 mm, left ventricular end-diastolic diameter 57 mm, right atrial long diameter 53 mm, right atrial transverse diameter 47 mm, right ventricular anterior-posterior diameter 23 mm, left ventricular ejection fraction 34%, pulmonary artery systolic pressure 44 mmHg, left ventricular mid- and lower-segment thrombus (approximately 36 × 34 mm; Figure 2A), decreased left and right ventricular wall movement, decreased left ventricular systolic and diastolic function, decreased right ventricular systolic function, and a small amount of pericardial effusion. It did not detect any congenital structural abnormalities, valvular heart disease, cardiomyopathy, or pericardial disease.

Blood tests revealed the following: white blood cell count of 13.62 × 10^9^/L (reference range: 3.5–9.5 × 10^9^/L) with elevated eosinophils (4.75 × 10^9^/L; reference range: 0.02–0.52 × 10^9^/L). Myocardial injury markers included elevated troponin I (0.059 ng/ml; reference range: 0–0.034 ng/ml), CK-MB 17.49 U/L (reference range: 7.0–25.0 U/L), lactate dehydrogenase 557.00 U/L (reference range: 135–214 U/L), lactate dehydrogenase isoenzyme 178.57 U/L (reference range: 17–96 U/L), and hydroxybutyrate dehydrogenase 399.00 U/L (reference range: 72–182 U/L). Biomarkers for impaired cardiac function included an elevated B-type natriuretic peptide level of 538.0 pg/ml (reference range: 0–100 pg/ml). Inflammatory indicators showed slightly elevated procalcitonin (0.061 ng/ml; reference range: 0–0.05 ng/ml) and hypersensitive C-reactive protein (3.48 mg/L; reference range: 0–2.87 mg/L). The coagulation profile included elevated D-dimer (1.86 mg/L; reference range: 0–0.55 mg/L).

Echocardiogram revealed left ventricular thrombosis, suggesting a potential source for the cerebral infarction. We proceeded to determine the underlying cause of the thrombosis, which can be broadly categorized into ischemic and nonischemic cardiomyopathy. Acute myocardial infarction, particularly involving the anterior wall, is the most common cause of ventricular thrombosis in patients with cardiomyopathy. However, this was unlikely in this case due to the following factors: young age, absence of traditional coronary artery disease risk factors, no family history of early-onset coronary artery disease, lack of chest pain symptoms, and dynamic ECG measured mild ST-segment depression and T-wave inversion (without dynamic changes). Therefore, ischemic cardiomyopathy leading to embolism and takotsubo cardiomyopathy were less likely. For nonischemic cardiomyopathy, echocardiogram findings were consistent with dilated cardiomyopathy, which carries a 30%–40% risk of being familial (6). However, the patient lacks a family history of this condition. Moreover, myocarditis, a potential cause of dilated cardiomyopathy, can be further classified based on endocardial biopsy findings (lymphocytic, eosinophilic, granulomatous, or giant cell) (7). She reported no significant abnormalities on cardiac ultrasound examination one year prior.

While her recent history included an infection, it was not suggestive of fulminant or severe myocarditis. Therefore, further investigation was necessary to clarify the cause of the dilated cardiomyopathy, particularly considering her elevated eosinophil count on routine blood tests. Eosinophilic syndrome is independently associated with ventricular thrombus formation, and the investigation was focused on this possibility. Eosinophils store various biologically active molecules in their granules. When extensively activated, these eosinophil-derived (toxic) substances can damage nearby tissues, leading to local inflammation, cytotoxicity, thromboembolic complications, and/or fibrosis (8). Cardiac involvement by eosinophils can manifest as either acute fulminant myocarditis (acute necrotizing cardiomyopathy) or chronic restrictive cardiomyopathy (Loeffler's endocarditis) (9). Given this patient's situation, Loeffler endocarditis emerged as a strong possibility as it could explain both the impaired heart function and the ventricular thrombus. The gold standard for diagnosing Loeffler endocarditis is an endocardial muscle biopsy; however, this procedure is invasive and not readily accepted by patients. CMR offers an alternative, providing a non-invasive imaging biopsy that can reveal inflammation, apical thrombosis, and fibrotic granulation tissue (10). To further investigate the cause of her condition, CMR was performed on the fourth day of admission. The results suggested Loeffler endocarditis involving the left ventricular myocardium, with apical occlusion thrombus formation (Figures 2B,C).

Therefore, the reason for the increase in her eosinophil count remains unclear. Their absolute eosinophil count on two separate peripheral blood tests exceeded 1.5 × 10^9^/L, fulfilling the diagnostic criteria for eosinophilia (HE) despite not meeting the usual 6-month duration requirement in some cases of rapidly developing eosinophil-related organ dysfunction. HE can be classified into four main types (11). Hereditary HE (HEF) typically presents in childhood with a family history of the condition. Her lack of family history argues against this diagnosis. Secondary HE (HER) is associated with various conditions: (1) Allergic diseases: She denies allergies and has no history of allergic conditions; (2) Allergic skin diseases (e.g., bullous pemphigoid and herpetic dermatitis) (12): She has no skin lesions suggestive of these conditions; (3) Medications (e.g., β-lactams, ciprofloxacin, clozapine, carbamazepine, and anti-tuberculosis drugs): She has a clear medication history without recent use of these drugs; (4) Infectious diseases (commonly parasitic infections): Parasitic infections are more prevalent in tropical and subtropical climates; (5) Rheumatic diseases and autoimmune conditions: Rheumatic and autoimmune workup resulted in negative findings; (6) Eosinophilic lung diseases: She denied respiratory symptoms. Chest CT scan and serum levels of immunoglobulin E and Aspergillus-specific immunoglobulin were all normal, arguing against eosinophilic pneumonia; (7) Malignancy: Comprehensive malignancy screening was performed. Carbohydrate antigen 125 (CA-125) level was elevated (114.5 U/ml, reference range: 0–35.0 U/ml), while neuron-specific enolase (NSE) and other tumor marker levels remained within normal limits. Given the limitations of CA-125 specificity (e.g., menstrual cycle, pregnancy, and endometriosis) (13), human epididymal protein 4 (HE4) was measured. Normal HE4 levels suggested the CA-125 elevation was not clinically significant. Abdominal examination to assess possible digestive tract tumors was unremarkable. Although Hodgkin's lymphoma can also cause eosinophilia, a peripheral lymph node ultrasound showed no abnormal lymph node enlargement, making this diagnosis less likely; and (8) Other factors, such as chronic graft-vs.-host disease (Gleich disease) and lack a history of related diseases, and this patient presented with no clear secondary cause.

Therefore, a hematological malignancy with clonal eosinophilia, also known as HEN, was considered. On the fifth day of admission, a bone marrow biopsy was performed. Bone marrow biopsy demonstrated normal hematopoietic function of the erythroid, granulocyte, and megakaryocyte lineages, along with an increase in eosinophils. There was no significant proliferation of immature cells (CD34+ or CD117+), and no obvious abnormalities were observed in other stages. Chromosome karyotype analysis was normal. According to the 2016 World Health Organization classification of myeloid tumors, the main categories of hematological tumors causing eosinophil elevation are myeloid/lymphoid neoplasms with eosinophilia and rearrangements involving PDGFRA, PDGFRB, FGFR1, or PCM1-JAK2 fusion (14). Therefore, we first evaluated these genes using interphase fluorescence *in situ* hybridization technology. Analysis for PDGFRA PDGFRB, FGFR1, and PCM1-JAK2 genes revealed no abnormalities. After excluding these three types of HE, a diagnosis of idiopathic HE was established.

**Figure 3:**
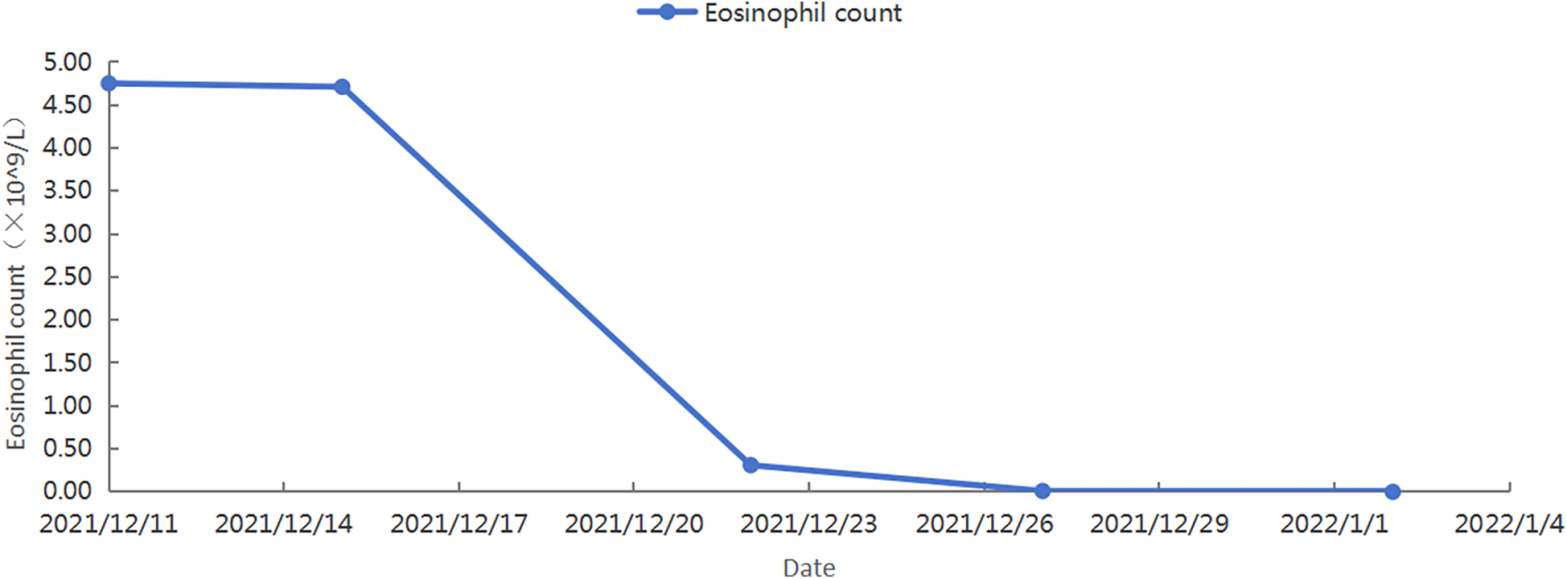
The patient's blood routine showed a gradual decrease in eosinophil count.

After identifying the cause, we developed a treatment plan. Currently, there are no clear evidence-based guidelines or consensus statements regarding the treatment of Loeffler's endocarditis. Existing case reports suggest that steroids are effective in some patients, while secondary immunosuppressants are effective in others. Steroid use was higher in patients with HES-induced steroid drugs (9). For patients with life-threatening eosinophilic complications, high-dose corticosteroids (at least 1 mg/kg of prednisone daily) are typically used for a short course (1). Therefore, we treated the patient with 80 mg of methylprednisolone for four days, followed by anticoagulation with 2.5 mg of warfarin. The patient's chest tightness and suffocation symptoms significantly reduced, and the amount of methylprednisolone gradually decreased. After one week of treatment, follow-up cardiac ultrasound was performed, showing an increased left ventricular ejection fraction of 50%. Following discharge, her treatment continued with oral warfarin and prednisone. Weekly INR monitoring ensured a range of 2.0–3.0. The prednisone dose was 50 mg with a reduction of 10 mg/week. After 20 days of treatment, follow-up cardiac ultrasound showed a normal left ventricular ejection fraction (54%). However, the left side of the heart remained enlarged. Additionally, a left ventricular thrombus was still present. Subsequently, multiple cardiac ultrasound examinations showed a decrease in the volume of the cardiac mass, although it did not completely disappear (Figure 2D). As expected, her eosinophil count gradually decreased after treatment, reaching 0 (Figure 3). However, the persistence of the cardiac mass led us to question its nature and we speculated that granulation tissue or other tissue types might be mixed with the thrombus. Therefore, the prednisone dosage was gradually reduced until discontinued, and imatinib was added. After one year of treatment with imatinib, the patient's cardiac mass completely disappeared (Figures 2E,F).

## 3 Discussion

To understand the nature of cardiac masses in Loeffler endocarditis, we reviewed literature reports from the past 20 years that utilized endocardial biopsy for confirmation. A case report described FIP1L1-PDGFR alpha fusion gene-positive myeloid leukemia in patients with eosinophilic syndrome affecting the heart and leading to Loeffler endocarditis. CMR revealed left and right ventricular masses, and pathology confirmed bilateral ventricular thrombosis and endocardial fibrosis (15). In 1994, Hussain et al. reported a left ventricular wall mass in a patient with Loeffler's endocarditis, confirmed as a thrombus by pathology (16). Strong et al. described a case of severe restrictive cardiomyopathy with refractory Loeffler's endocarditis symptoms, wherein the patient underwent heart transplantation, and the pathology of the transplanted heart revealed wall thrombosis (17). Chao et al. reported a patient with unexplained eosinophilic syndrome who developed a right ventricular mass. Despite treatment, the condition worsened, and the autopsy revealed ventricular fibrosis and multiple thromboses (18).

However, limitations exist in our understanding. These endocardial biopsy results are primarily obtained from autopsies or heart transplants due to the invasive nature of the procedure, often performed in late disease stages. Another study reported about an asymptomatic patient with a history of eosinophilia for 3 years, who was found to have a heart mass during a physical examination (19). Echocardiography showed an uneven mass measuring 30 × 39 mm in the left ventricle, and CMR strongly suggested malignant cardiac tumor invasion of the papillary muscle. Pathological examination after surgical resection confirmed mixed thrombus and irreversible myocardial damage caused by eosinophil infiltration, which had progressed to late-stage Loeffler endocarditis (19). Gudmundsson et al. reported a case of Loeffler endocarditis wherein transesophageal echocardiography identified a soft-tissue mass on the right ventricular septum (20). Surgical exploration revealed a large mass involving the right coronary sinus and aortic valve lobule. Pathology showed eosinophilic infiltration and fibrosis, but no thrombus (20). Moreover, Corradi et al. reported an early endocardial biopsy in Loeffler's endocarditis, revealing granulomatous lesions rich in eosinophils within the subendocardial myocardium (21).

While most reported cases of Loeffler endocarditis demonstrate a thrombus as the cardiac mass, the late-stage nature of biopsies in many cases is a limitation. Loeffler's endocarditis progresses through stages of acute necrosis, thrombus formation, and fibrosis (18). The composition of the cardiac mass likely varies depending on the disease stage, potentially involving multiple components simultaneously. Researchers propose that cerebral infarction in idiopathic HES patients might be caused by thromboembolism due to endocardial myofibrosis or eosinophil-mediated endothelial toxicity (22). We suspected the former to be the cause of our patient's symptoms. If imaging in the early stages cannot determine the nature of the endocardial vegetation, should anticoagulants be used? Studies suggest that anticoagulation can improve long-term ventricular remodeling by preventing thrombus formation (23). Additionally, if thrombosis is present, anticoagulant therapy can prevent pulmonary or systemic embolic events and thrombus growth (2). Therefore, immediate anticoagulation after a Loeffler endocarditis diagnosis is recommended, regardless of confirmed thrombosis.

While HES symptoms are diverse, with fatigue (26%), cough (24%), difficulty of breathing (16%), myalgia or vascular edema (14%), rash or fever (12%), and rhinitis (10%) being common (24), cardiac involvement is often rare and has non-specific clinical signs. Loeffler endocarditis primarily manifests through non-specific symptoms like heart failure, chest pain, and arrhythmia, mimicking other conditions. Clinicians might be unfamiliar with Loeffler endocarditis, leading to misdiagnosis as more common conditions like myocarditis or acute coronary syndrome. Though tissue biopsy remains the definitive test for confirming myocardial eosinophilic infiltration, its invasive nature discourages patients and limits its use. Moreover, not all hospitals have the capability to perform endocardial biopsies, further hindering early diagnosis and timely treatment. Clinicians should comprehensively evaluate the patient's condition and rule out reasons other than eosinophilia for organ dysfunction.

An absolute eosinophil count (AEC) greater than 0.5 × 10^9^/L defines HE, while HES is diagnosed with an AEC of 1.5 × 10^9^/L or higher. In patients with persistent or recurrent eosinophilia, tissue infiltration by eosinophils and the release of eosinophil-derived mediators and cytotoxic proteins can lead to clinically relevant organ damage, resulting in eosinophilia syndrome (8). The goal of treatment is to alleviate eosinophil-mediated organ damage, which becomes crucial when considering factors that predict poor prognosis, such as concurrent myeloproliferative syndrome, corticosteroid-refractory HE, heart disease, male sex, and eosinophil counts. Therefore, intervention is not necessary for mild eosinophilia (less than 1.5 × 10^9^/L) without symptoms or signs of organ involvement. However close monitoring is essential. Doctors should pay close attention to eosinophilia, even if the initial count is normal but progressively increases during hospitalization. Prompt investigation of potential organ involvement and early identification of the cause is crucial for reducing mortality rates and avoiding overdiagnosis and unnecessary treatment.

Despite ongoing research, clear conclusions about treatment and prognosis for HES and Loeffler endocarditis remain elusive. For most newly diagnosed patients with HES, besides those with eosinophilia secondary to myeloid tumors, corticosteroids are the first-line treatment. While the mechanism of action is not fully understood, initial response rates are high (around 85% after a month). However, long-term use can lead to treatment resistance or significant side effects in many patients (1). Several promising new biological therapies targeting eosinophil maturation factors, such as interleukin (IL)-5 and its receptor, or IL-4/IL-13, are emerging in clinical practice (25). Tailoring medication regimens to individual patient characteristics is crucial to optimize treatment effectiveness and minimize side effects. Long-term follow-up is essential for all patients.

## Data Availability

The raw data supporting the conclusions of this article will be made available by the authors, without undue reservation.
